# Designing interfaces for digital physical ability self-assessment: a user-centered iterative approach

**DOI:** 10.3389/fdgth.2026.1815892

**Published:** 2026-06-25

**Authors:** Agnieszka Jaff, Mia Folke, Annica Kristoffersson

**Affiliations:** Department of Computer Science & Engineering, Mälardalen University, Västerås, Sweden

**Keywords:** digital exercise tools, exercise-based assessment, physical ability, physical activity self-assessment tool, physical function assessment, usability, user interface (UI) design

## Abstract

**Background:**

Digital physical ability self-assessments offer an accessible alternative to resource-intensive objective assessments, but their outcomes depend on how well user interfaces (UIs) support correct exercise execution and self-assessment.

**Methods:**

This study examined how UI design influences users' interpretation, execution, and self-assessment when using a digital physical ability test. Adopting an iterative, user-centered design approach, six prototype versions were developed and evaluated across four usability phases. Twenty-four working-age adults participated in think-aloud usability tests while performing a single instructionally complex test exercise (Tempo-guided chair squat). Video-recorded sessions were qualitatively analyzed to identify recurring usability breakdowns and examine how UI design shaped participants' self-assessments in relation to researchers' assessment across design iterations.

**Results:**

Iterative usability testing revealed recurring breakdowns in how participants interpreted and acted on the test exercise instructions. Key usability issues included misinterpretation of tempo cues, unclear boundaries between correct and incorrect execution, loss of repetition-count awareness, insufficient visual support during execution, overlooked safety-critical setup information, interface inconsistencies, misunderstanding of exercise-relevant concepts, and misaligned self-assessment criteria. These issues led to systematic execution errors and misjudgments of execution quality across prototype versions.

**Conclusion:**

The findings conceptualize digital physical ability self-assessment as a multi-layered interaction that places sustained cognitive and physical demands on users. The study contributes a set of transferable design principles describing the interactional support required to enable accurate physical-ability self-assessment. While grounded in a specific context, these design principles may offer insights for exercise-based UIs more broadly, although their generalizability requires further validation.

## 1 Introduction

Self-assessed physical function refers to individuals' personal evaluation of various aspects of their physical capabilities and fitness (1). A common example is a questionnaire in which people rate their own ability in different domains of physical performance (2). Health-related physical fitness comprises multiple dimensions, including endurance, strength, flexibility, balance, and coordination (3) which together contribute to the individuals' physical ability.

Objective assessment of physical ability in healthcare settings is often resource-intensive and typically limited to individuals with identified risks, such as those having fallen or managing conditions affecting physical functioning (4). As a result, healthy working-age adults—despite being a key target group for physical activity promotion and early detection of weaknesses in physical abilities—are rarely assessed in routine healthcare practice. Consequently, there is a growing need for tools that enable self-administered assessment of physical abilities without requiring professional supervision. Digital health and fitness apps that incorporate self-assessment data can support personalized guidance and adaptive training programs, which have been shown to be feasible and acceptable (5), and also leading to significant improvements in physical function (6).

However, ensuring that users can clearly understand and appropriately self-assess physical abilities remains a major challenge. Inappropriate or poorly designed assessment methods can produce misleading or unreliable results (7). Self-report approaches—such as questionnaires, diaries, or logs—are advantageous in terms of accessibility, low burden for the respondent, and cost, but suffer from limited reliability and validity due to recall errors and subjective bias (7). These limitations underscore the need for more precise, technology-supported methods that combine the accessibility of self-assessment with features that support consistent and informed user judgements. Developing valid and reliable digital tools is an important step toward achieving more accurate self-assessment outcomes in physical activity contexts (8).

For digital exercise-based self-assessments, the user interface (UI) plays a crucial role in supporting correct test execution and maintaining user engagement. Self-assessments must be easy to understand and clearly convey how the test exercise should be executed (8). Poorly designed UIs can lead to confusion, reduce motivation, and compromise data quality. Misinterpretation of instructions and under- or overestimation of execution quality may result in discrepancies between perceived and actual exercise execution, limiting the reliability of self-assessments. Errors in exercise execution not only undermine measurement validity (7) but may also increase the risk of injury if performing the movement incorrectly (9).

Designing effective exercise UIs is therefore a demanding task that requires careful attention to usability. Usability is not a single property, but a multidimensional concept which can be described through five key attributes: learnability, efficiency, memorability, errors, and satisfaction (10). A usable system should be easy to learn so that users can quickly start using it, efficient to use once learned, and easy to remember even after periods of non-use (10). It should minimize errors, allowing users to recover easily when they occur, and it should be pleasant and satisfying to use (10). Usability testing helps prevent real-world mistakes (11), reduces the need for extensive instructions and support, and increases acceptance and engagement by ensuring the technology feels practical, intuitive, and enjoyable to use (12).

Established UI design principles offer important guidance for supporting usability and interaction (e.g., 10, 13, 14), yet their application to complex, real-world contexts is not always straightforward. In particular, ensuring that instructional content is interpreted as intended during exercise requires moving beyond general UI design principles toward iterative, user-centered design (UCD). UCD emphasizes early and continuous involvement of end users throughout the system development process. Iteration is a central principle of the UCD approach, which means that successive versions of the system are repeatedly tested and refined based on user feedback (15, 16). Iterative methods are widely used in health technology design to enhance usability, accessibility, and user acceptance (17, 18). Through iteration, real-world usability challenges can be identified, enabling researchers to refine and enhance the design to increase effectiveness and adoption (12, 19). A well-designed UI not only facilitates intuitive interaction but also directly influences users' engagement (20).

Several studies have explored feasibility, reliability and usability of tests assessing physical abilities delivered through digital or smartphone-based systems both in self-administered formats (21–25) and supervised (26). While these studies generally report acceptable feasibility and reliability under controlled or supervised conditions, they also highlight persistent challenges related to instruction comprehension, execution accuracy, and technology handling during unsupervised use. Importantly, much of the prior work has focused on older adults and clinical or rehabilitation populations. Less is known about how healthy working-age adults interpret and self-assess physical abilities when relying solely on a digital UI, particularly in relation to the different UI design elements.

We have developed six interactive prototypes for digital self-assessment of physical ability related to fall risk as part of the PRE-fall research project. The intended use context of this self-assessment is to evaluate users' physical abilities and to use this information to deliver personalized guidance and adaptive training programs in a single tool. The prototypes represent different design versions that were tested with users to inform iterative improvements. Each prototype includes several test exercises. The complete set of test exercises and their role in assessing weakness in different physical abilities are described elsewhere (27).

In this study, we focus exclusively on the UI design of one test exercise within this set, selected for its instructional complexity. The aim was to examine its usability and feasibility from the perspective of its intended users (working-age adults) through an iterative, UCD process. Across four usability phases, the study explored how users perceived and interpreted successive versions of the test exercise UI and how usability-related execution issues emerged during execution and self-assessment. Observed issues were used to inform progressive UI refinement and were synthesized into design-relevant insights for digital physical activity self-assessment tools, rather than to develop a finalized interface solution.

Beyond contributing to the design of digital physical ability self-assessment tools, this study may offer broader implications for exercise-based instructional UIs. By examining how users understand, follow, and enact digitally presented test exercise instructions, the findings provide transferable insights into designing exercise-related interactions that support correct interpretation, execution, and user confidence. The study also advances the understanding of users' experiences when engaging with digital exercise tools.

The remainder of this article is structured as follows. Section 2 describes the study design, prototypes, procedure, ethical considerations and data analysis. Section 3 presents the results from the usability testing. Finally, Section 4 discusses the findings in relation to usability in physical ability self-assessment and outlines their implications in the form of transferable design principles, followed by limitations, future work, and conclusions.

## 2 Methods

This section is organized as follows. Subsection 2.1 describes the study design. Subsection 2.2 details the data collection procedures. Subsection 2.3 addresses ethical considerations. Subsection 2.4 describes the data analysis process performed.

### 2.1 Study design

**Figure 1:**
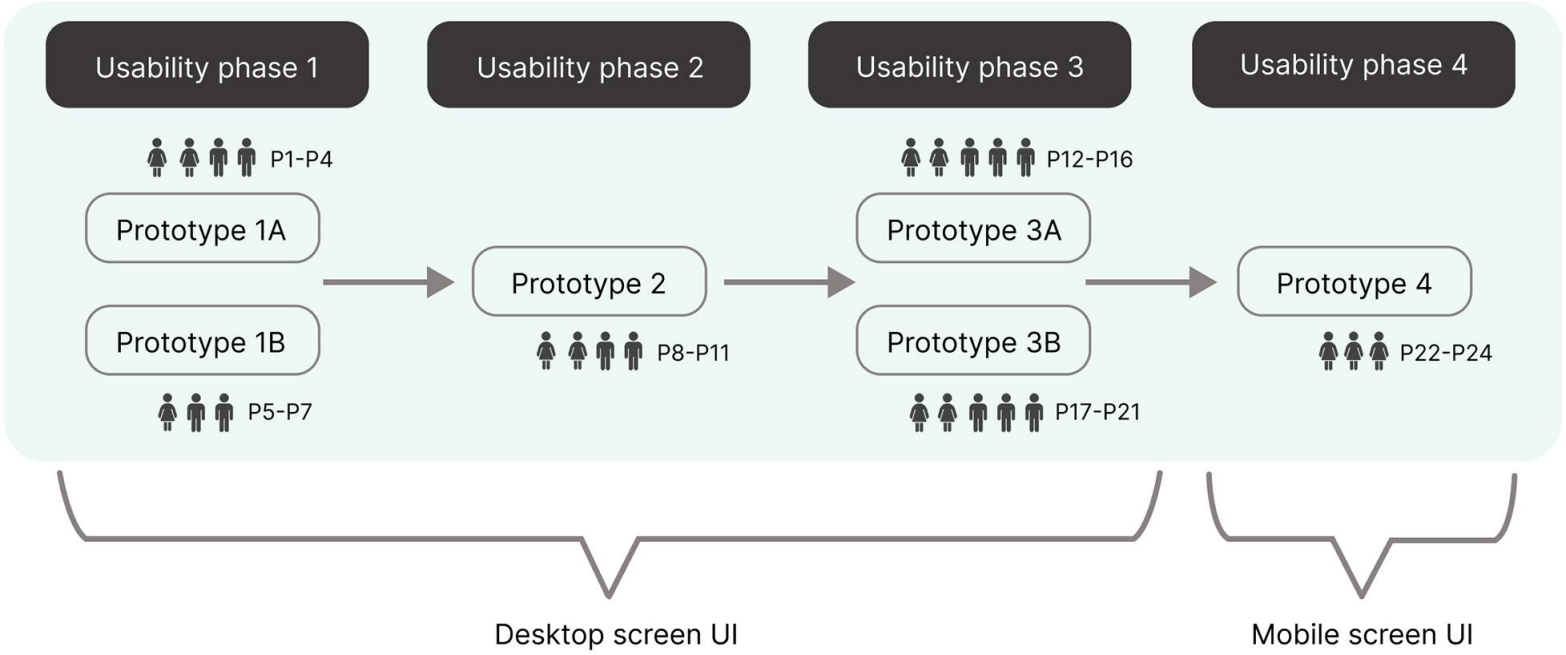
Overview of the iterative usability testing process. After each usability phase, the design was refined according to the participants' feedback and observations. Person symbols show the number of participants and their sex. Participants are labelled P1–P24.

The study followed an iterative, UCD process and started in March 2023. Across four usability phases, six prototypes were developed and tested, with each usability phase informing subsequent design decisions (see Figure 1 for an overview of the usability phases, prototype progression, and participant distribution). Rather than aiming for exhaustive identification of all possible usability issues within a single test period, the study adopted an iterative testing strategy in which small user samples were used to identify design-relevant usability issues and guide successive refinement, consistent with established usability testing practices (10, 28). Although the prototypes included multiple test exercises (27), the analysis in this article focuses on a single test exercise—the *Tempo-guided chair squat*—as a design case within the UCD process.

The *Tempo-guided chair squat* was selected for its instructional complexity involving the use of equipment, and a description of movement according to set criteria at three metronome-guided movement tempos. This complexity provides a strong case for usability and feasibility examination of instruction comprehension, movement execution, and self-assessment within a digital test UI. The test exercise shares similarities with the 1-min sit-to-stand test which is used to assess exercise capacity, and which is particularly fitted to be performed when time and space are limited (29). It also shares similarities with the test 5-repetitions chair rise demonstrating excellent reliability in the context of fall risk assessment (26). In both the 1-min sit-to stand test and the 5-repetitions chair rise test, the test subject is required to sit down on the chair in between each of the chair rises. We assessed the participants' ability to perform three squats quickly at a steady tempo without sitting down. The *Tempo-guided chair squat* was designed to incorporate three metronome-guided tempos to support the assessment of the level of weakness in the corresponding physical ability. This design choice was informed by prior observations indicating that users in unsupervised settings often struggle to reliably count repetitions. The design aims to reduce this cognitive burden while enabling self-assessment at faster, standardized tempos in an unsupervised digital setting.

A total of 24 participants were recruited through convenience sampling via academic networks and personal contacts. Participants were eligible for inclusion if they were working-age adults of any sex and speaking Swedish.

As illustrated in Figure 1, the first three usability phases focused on a low-fidelity desktop-based UI, starting with two parallel early versions (Prototypes 1A and 1B), followed by a consolidated design (Prototype 2), and later two refined versions (Prototypes 3A and 3B). The final usability phase evaluated a high-fidelity mobile screen version (Prototype 4). While the desktop format was suitable for early-stage usability exploration and rapid iteration, the transition to a mobile version was driven by the intended use context of the tool, as mobile devices are portable and embedded in everyday life, enabling use across a range of real-world contexts, including physically active situations (30).

The initial reliance on text and simple figures in Prototypes 1–3 was a deliberate choice aligned with iterative, user-centered design principles, where early-stage low-fidelity prototypes prioritize simplicity to enable rapid exploration and testing of core design ideas before introducing more complex and resource-intensive elements (10, 13). The initial usability phases enabled iterative refinement of both the content and wording of instructions, revealing ambiguities, missing elements and misinterpretations through user interaction. Building on these insights, Prototype 4 incorporated video-based instruction, aligning with multimedia learning principles, which suggest that combining visual demonstration with spoken instruction can enhance understanding of complex tasks (31). Importantly, textual and visual instructions were retained alongside video instructions in Prototype 4, allowing users to engage with different forms of instruction depending on their preferences and individual needs, consistent with principles of interaction design (13). In addition, the evolving design was informed by established usability principles, particularly consistency and standards, visibility of system status and recognition rather than control (32), which guided decisions related to layout consistency, feedback, and the placement of interactive elements to support low cognitive load (33).

The decision to test either one or two parallel prototypes depended on whether multiple alternative design solutions required empirical comparison before consolidation. Each prototype version was tested with 3–5 participants. In Prototypes 1–3, the metronome used in the *Tempo-guided chair squats* was not embedded in the UI. As the prototypes were tested by Swedish-speaking adults, all content was originally produced in Swedish. For this article, the content has been translated into English. Number of participants, their labels, and sex are illustrated in Figure 1.

Each usability phase included usability testing combined with the think-aloud technique, a verbal report method that enables insight into users' cognitive processes as they occur during interaction. By capturing participants' concurrent reasoning and reactions, the method is particularly valuable for identifying usability issues and understanding how users interpret and solve tasks within the system (34). After each usability phase, participants' verbalized reactions and reasoning during the tests and systematic observations derived from video recordings were translated into concrete design requirements, which directly informed subsequent prototype versions. This iterative process ensured that design decisions were continuously informed by usability findings, aligning with established guidelines for UCD (10, 28). Design modifications were introduced based on identified usability issues that represented breakdowns in task completion, explicit user confusion, or recurring interaction difficulties observed across participants. Issues were prioritized for redesign when they affected core task execution or the validity of self-assessment outcomes.

The adequacy of each prototype version was assessed based on whether previously identified usability breakdowns were resolved or mitigated, and whether new issues emerged during usability testing. Usability phases were continued for as long as critical breakdowns affecting task execution or self-assessment quality were observed. In the final usability phase, newly identified issues were of lower severity and did not substantially compromise task performance, indicating that further refinement would unlikely result in substantial improvements within the scope of this study.

Consequently, Prototype 4 is not presented as a finalized solution, but as the most refined prototype version within the scope of this study. It represents a stage at which key design directions had stabilized, and major usability breakdowns had been sufficiently identified and addressed to support the extraction of design insights. Further refinement is addressed as part of future work.

### 2.2 Procedure

At the start of each session, participants were asked to interact with the prototype, interpret its content independently, perform the test exercises, and verbalize their reactions and reasoning throughout the session. Participants were also informed that the purpose was to evaluate the prototype rather than their own understanding of its content. To account for iterative changes across prototypes, phase-specific test scripts were used in each usability phase to introduce and guide participants through the session (Supplementary Material S1). The sessions were video recorded for subsequent analysis and comparison across participants and prototype versions.

Researchers emphasized during the sessions that the UI shown in Prototypes 1–3 was a low-fidelity, non-interactive prototype. Participants were therefore asked to describe aloud where they would click, write, or navigate rather than interacting directly with the UI. In Prototype 4, participants used a high-fidelity and interactive mobile phone version and were able to interact with the prototype directly. During tests of Prototype 4, screen recording software was used to record participants' interactions with the UI for later analysis. As in earlier phases, participants were instructed to verbalize their thoughts throughout the session, including when assessing their test exercise performance.

From Usability phase 3 onward, participants were asked to perform all the test exercises in the prototype. For instance, when exercises required testing both the left and right leg, participants tested both rather than only their preferred leg, as in earlier usability phases. Following the completion of the full test, test exercises for which participants' self-assessments differed from the researcher's assessment were repeated. During these retests, participants were also asked to explain how they had interpreted the UI using the think-aloud method.

The sessions were conducted in a room located within university premises. The room was equipped with a table on which a laptop displaying the prototype was placed during sessions involving a desktop screen UI (Usability phases 1–3), along with all equipment needed to perform all the test exercises. The setup provided sufficient space for participants to comfortably complete all test exercises. During sessions involving the mobile screen UI (Usability phase 4), no phone holder or stand was available. Participants chose to interact with the mobile device by holding it in their hands or by placing it on the table while performing the test exercises.

The sessions were conducted with one to three researchers present. Researchers adopted a fly-on-the-wall observation approach, writing only minimal notes and refraining from providing instructions or guidance. This was done to maintain validity of the sessions and to minimize researcher influence on participants' interpretations and interaction behaviors. Intervention occurred only when necessary to prevent potential injury.

### 2.3 Ethical considerations

All participants received written and oral information about the study and informed consent in writing was obtained from each participant prior to commencing the study. Data were pseudonymized, securely stored, and all identifying information was eliminated to ensure confidentiality. Although the usability tests were video recorded, the camera was positioned to avoid capturing participants' faces. However, the participants were informed that incidental facial recording could occur in some instances. Participation was voluntary, and participants were informed of their right to withdraw at any time without consequence. The study was conducted in accordance with good research practice and approved by the Swedish Ethical Review Authority (2022-06690-1). No compensation was provided for participation in this study.

### 2.4 Data analysis

Across all usability phases, the usability evaluation focused on learnability, efficiency, and errors, as these aspects are most critical in early-stage design and directly influence the feasibility of physical ability self-assessments. Findings from each usability phase were analyzed qualitatively through repeated review of video recordings and participants' think aloud verbalizations and integrated into the next design iteration to address usability issues and enhance clarity and functionality of the prototypes.

The analysis followed an inductive approach, where usability issues were identified from the data without predefined coding categories. The video recordings were independently reviewed by all three authors, who annotated observed events (e.g., breakdowns in task completion) and participants' verbalizations (e.g., expressions of confusion), along with representative quotations related to usability issues. The main author subsequently consolidated and synthesized these annotations into a set of usability issues. Identified usability issues were iteratively documented for each prototype version, grouped into recurring patterns based on frequency and similarity across participants and prototypes, linked to specific UI elements and tracked across usability phases. Relevant quotations were selected to illustrate identified usability issues.

Additionally, once all usability phases were completed, a cross-phase analysis of the full set of video recordings was analyzed to examine how participants reasoned about their own test exercise execution. For this purpose, participants' self-assessments were compared with researchers' assessment of the test exercise execution by reviewing the video recordings and noting whether the participants' reported performance aligned with researchers' observed tempo adherence and overall movement execution as described in the test instructions. This comparison was used to identify recurring patterns and mismatches across all tested prototype versions and to explore how identified usability issues and UI shaped participants' assessments.

Moreover, to move beyond prototype-specific observations, usability findings were synthesized into a set of design principles in the discussion.

## 3 Results

This section is organized as follows. Subsection 3.1 provides information on the participants. Subsections 3.2–3.7 present findings structured around the prototype versions (1A, 1B, 2, 3A, 3B and 4). Subsection 3.8 presents observed patterns related to self-assessment during test exercise execution. Subsection 3.9 provides an overview of all identified usability issues across iterations.

### 3.1 Participants

Across all design iterations, 24 participants took part (12 women, 12 men), aged 29–63 years. The sample was predominantly highly educated and mainly engaged in office-based work. Full demographic details are provided in Table 1.

**Table 1:** Participant demographics.

Characteristic		*n* = 24
	Mean age (years)	48.63
	Standard deviation (years)	8.36
Sex		*n* (%)
	Women	12 (50)
	Men	12 (50)
Education		*n* (%)
	Higher education	22 (91.67)
	Upper secondary	2 (8.33)
Occupation		*n* (%)
	Office-based	23 (95.83)
	Manual labor	1 (4.17)

### 3.2 Prototype 1A

**Figure 2:**
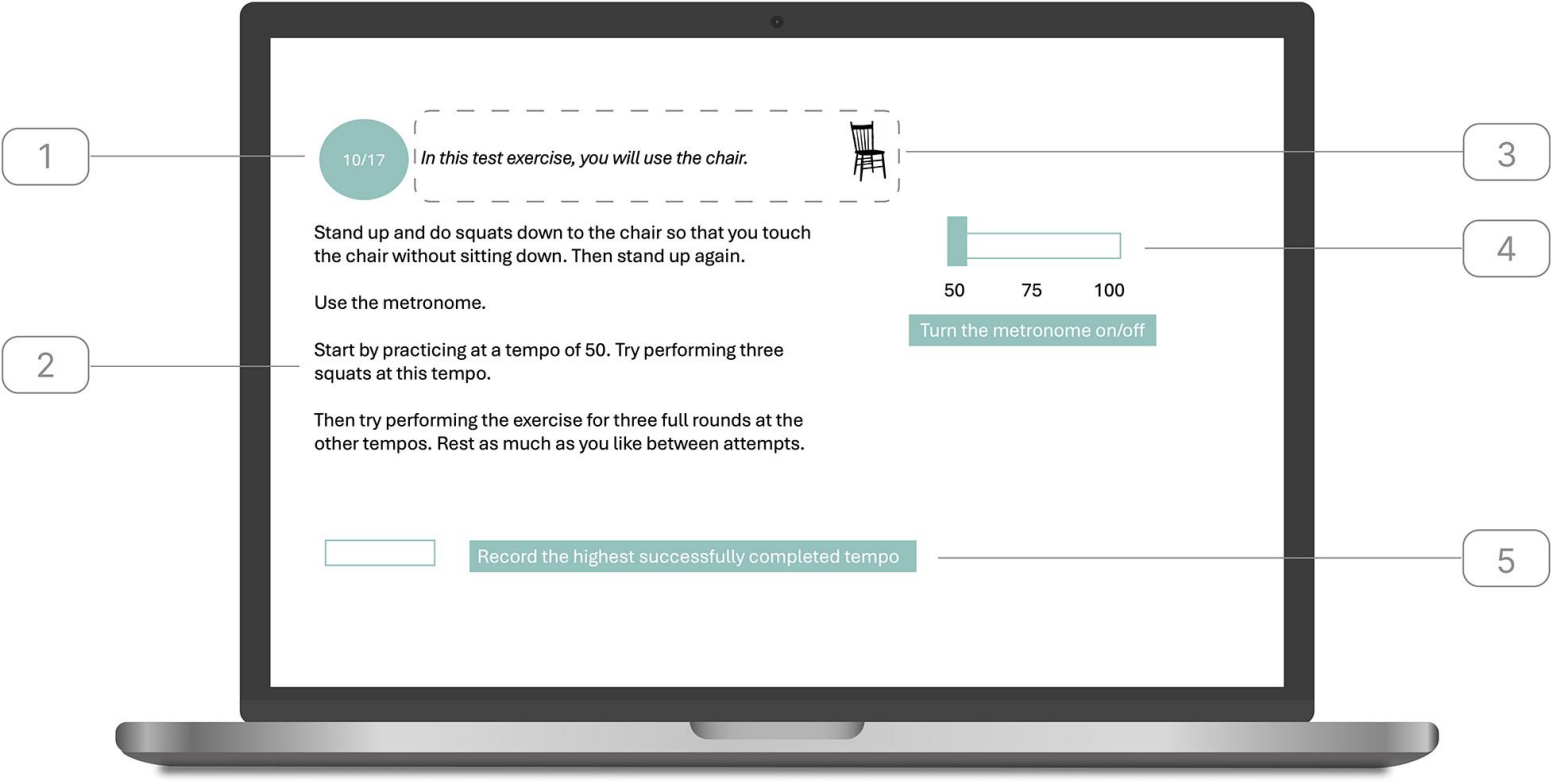
Screenshot of the test exercise instruction in Prototype 1A: (1) Numeric progress marker. (2) Text instruction. (3) Required equipment. (4) Metronome control slider. (5) Input field with a button to record the highest completed tempo.

**Figure 3:**
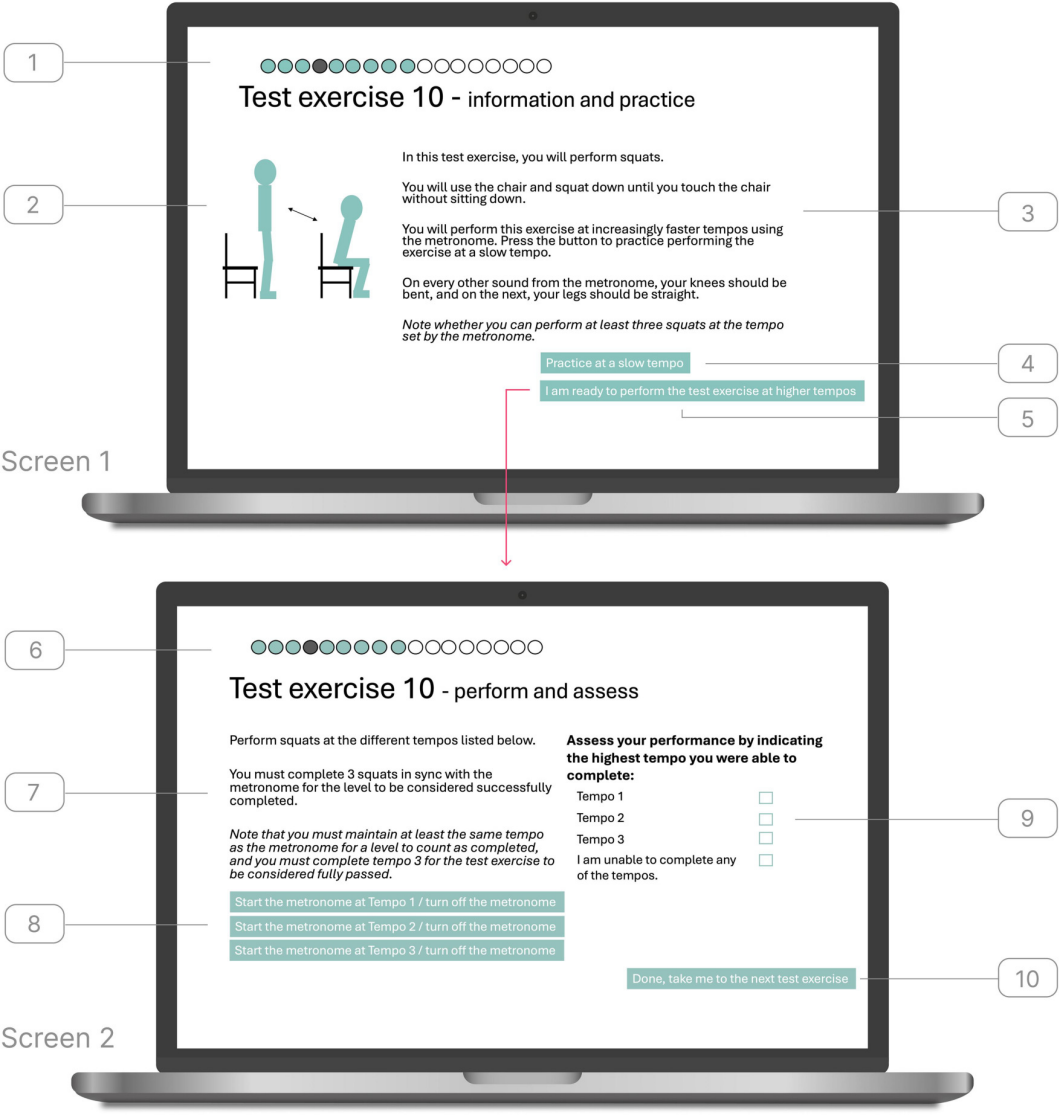
Screenshots of the test exercise instruction in Prototype 1B: Screen 1: “Test exercise 10—information and practice”: (1) visual progress marker. (2) Movement illustration. (3) Text instruction. (4) Button to start a 50 BPS metronome for slow practice. (5) Button that advances to the next screen. Screen 2: “Test exercise 10—perform and assess”: (6) Visual progress marker. (7) Text instruction. (8) Three tempo selection buttons. (9) Self-assessment section with tempo-based checkboxes. (10) Button that advances to the next test exercise.

**Figure 4:**
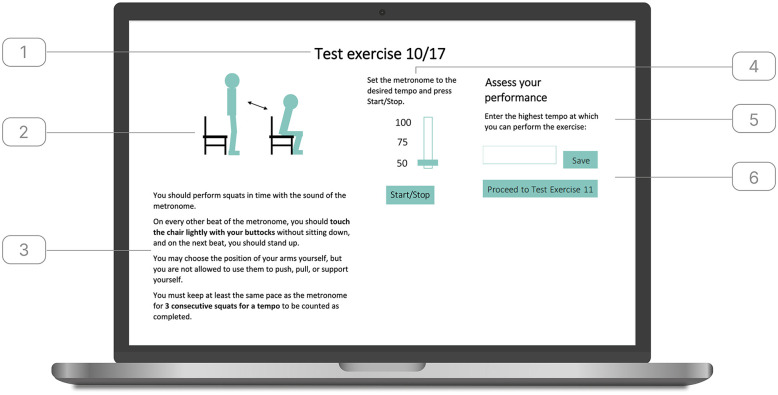
Screenshots of the test exercise instruction in Prototype 2: (1) Header and a numeric progress marker. (2) Movement illustration. (3) Text instruction. (4) Metronome control slider. (5) Input field to record the highest completed tempo. (6) Button that advances to the next test exercise.

**Figure 5:**
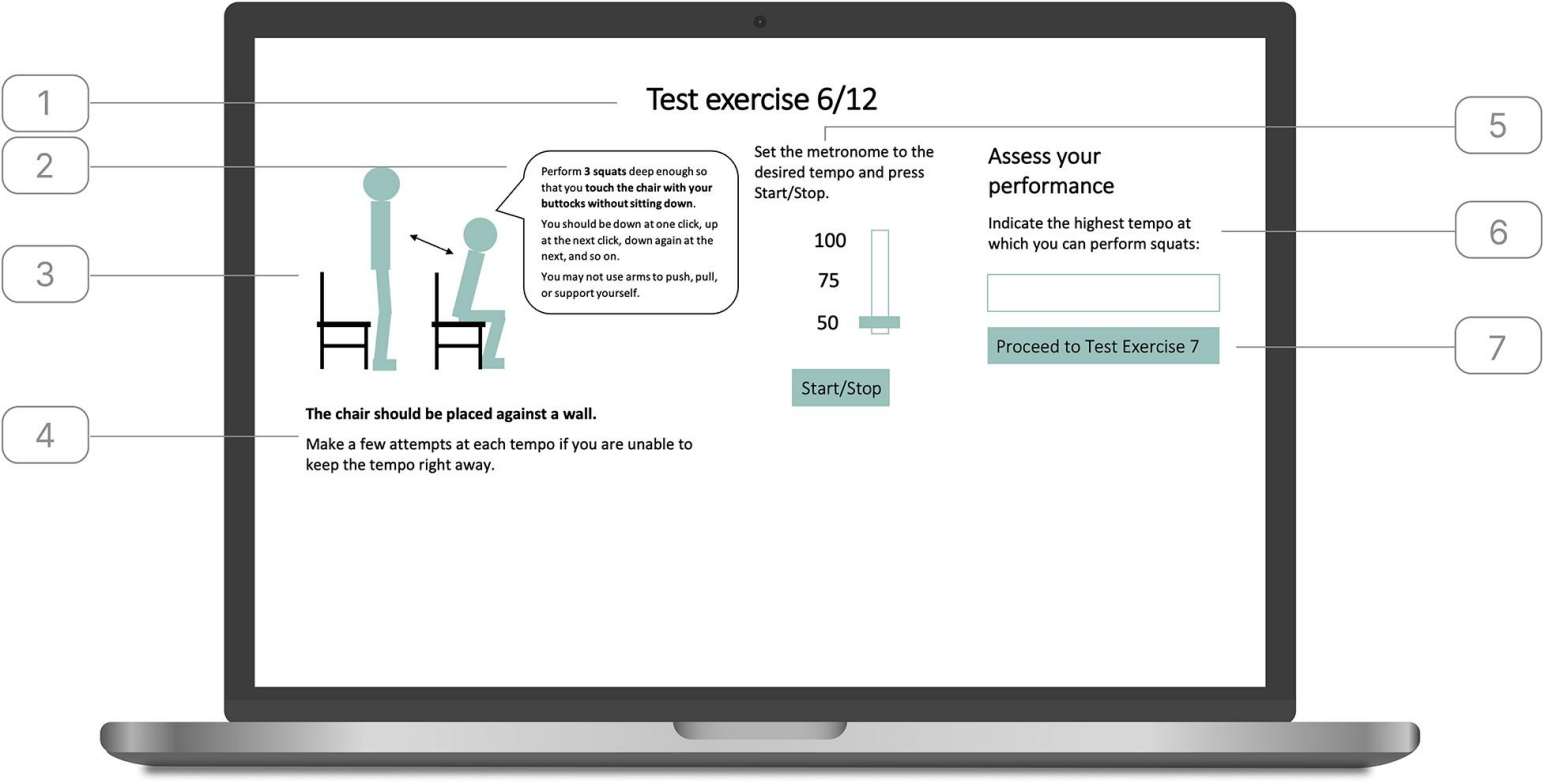
Screenshots of the test exercise instruction in Prototype 3A: (1) Header and a numeric progress marker. (2) Text instruction. (3) Movement illustration. (4) Additional text instructions. (5) Metronome control slider. (6) Self-assessment section with an input field. (7) Button that advances to the next test exercise.

Prototype 1A (Figure 2) presented the test exercise instruction on a single screen with a numeric progress marker indicating overall progression (Figure 2-1). The test exercise was explained through a text instruction (Figure 2-2), supported by a chair illustration with accompanying text indicating that a chair should be used (Figure 2-3). A horizontal metronome control allowed selection of 50, 75, or 100 beats per minute (BPM) via a slider and included a button to turn the metronome on or off (Figure 2-4). The screen also included a self-assessment input field with a corresponding button for recording the highest successfully completed tempo before advancing to the next test exercise (Figure 2-5).

#### 3.2.1 Usability issues identified in Prototype 1A

The phrase “three full rounds” was interpreted with varying confidence. Three participants (P1, P2 and P3) confidently equated it with performing three squats, while one (P4) completed the correct number of squats but expressed hesitation about whether she had understood the instruction correctly.

Another usability issue identified was related to visual clarity. The lone chair illustration led all four participants to guess how far to stand from the chair or how deep to squat. Without a body illustration, everyone (P1-P4) performed squats too shallowly.

P2 expressed uncertainty about what a squat is, stating “A squat? How do you do it?” and yet performed the correct movement.

Arm placement was unclear for P3 who meant that further instructions are desired, expressing: “I didn't know what to do with my arms”. Unlike earlier balance-testing exercises in this prototype, this test exercise provided no explicit guidance on arm placement.

#### 3.2.2 Key design implications

After usability testing of Prototype 1A, the identified issues signaled a need for improvement in the next iteration (Usability phase 2).

Ambiguous terminology—such as the phrase “three full rounds”—indicated a need for clearer terms. The lack of a test exercise illustration signaled a need to visually convey the squat movement rather than depicting the chair alone. Findings also indicated a need for a body illustration with clearer visual cues and explicit arm-placement guidance. At the same time, the design needed to maintain brevity by relying on minimal visuals and persistent cues, such as a simple figure showing correct body position and a clear movement indicator.

### 3.3 Prototype 1B

**Figure 6:**
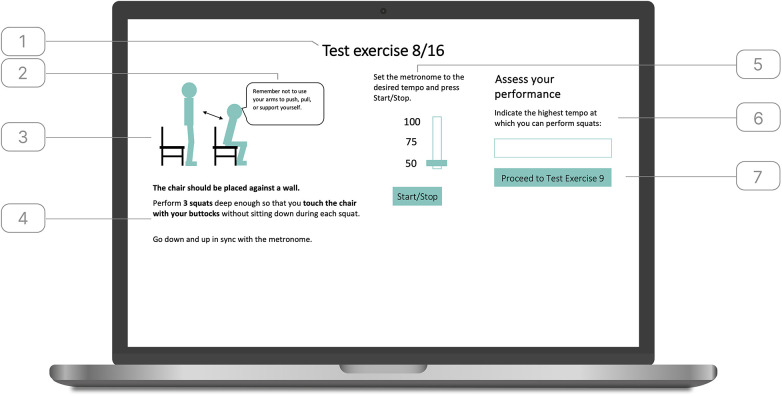
Screenshots of the test exercise instruction in Prototype 3B: (1) Header and a numeric progress marker. (2) Prohibited arm use instruction. (3) Movement illustration. (4) Text instruction. (5) Metronome control slider. (6) Self-assessment section with an input field. (7) Button that advances to the next test exercise.

**Figure 7:**
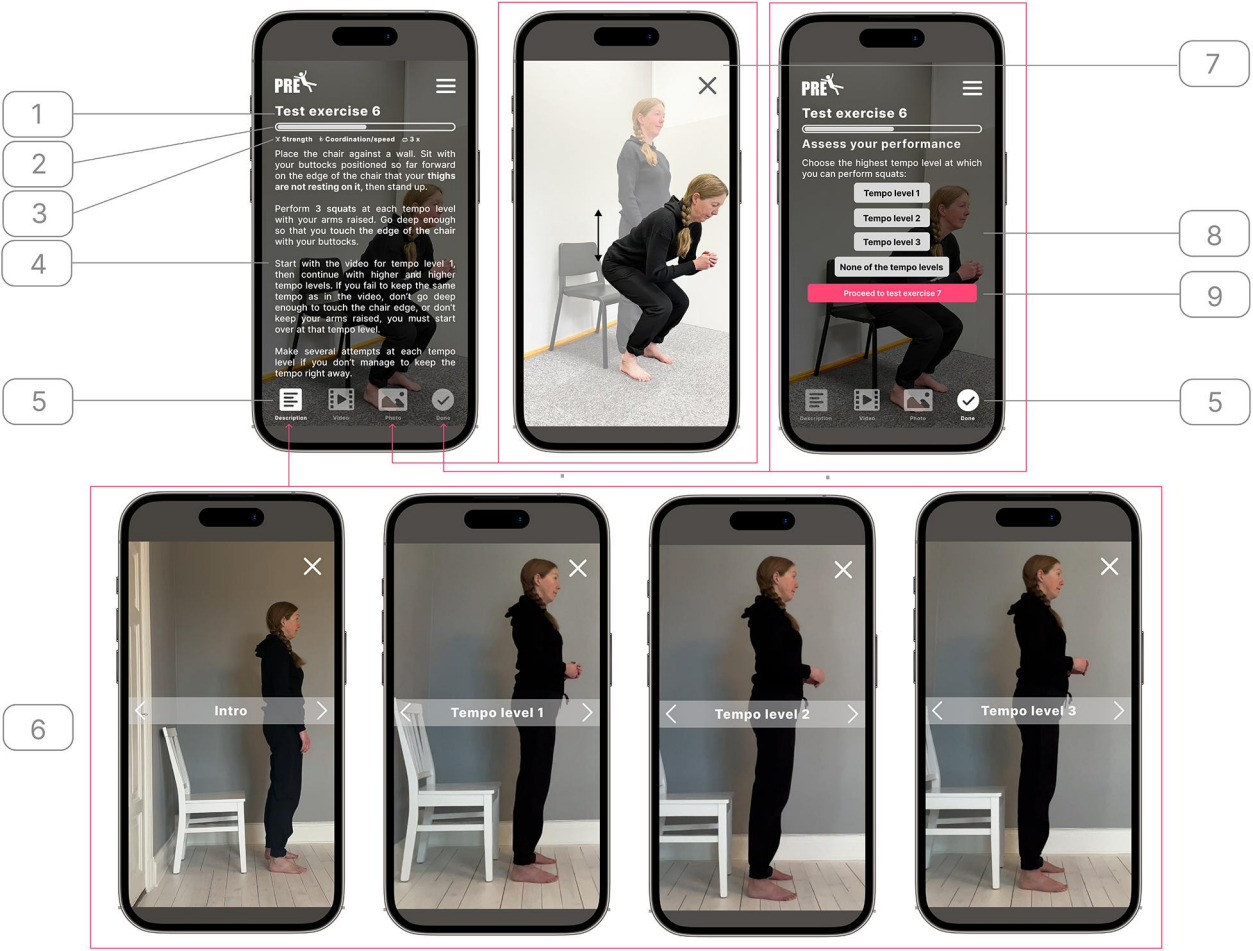
Screenshots of the test exercise instruction in Prototype 4: (1) Header. (2) Visual progress marker. (3) Icons representing physical abilities tested and repetitions. (4) Text instruction. (5) Bottom navigation panel. (6) Video instruction section. (7) Photo instruction. (8) Self-assessment section. (9) Button that advances to the next test exercise.

Prototype 1B (Figure 3) presented the test exercise instruction across two screens. The first screen combined a visual progress marker displaying completed and remaining steps (Figure 3-1), a simple stick-figure illustration of the test exercise movement (Figure 3-2), and a text instruction describing the test exercise (Figure 3-3), together with a button that started a metronome at 50 BPM for slow practice (Figure 3-4) and a button that advanced to the next screen for testing at higher tempos (Figure 3-5). The second screen continued to display the visual progress marker (Figure 3-6), added further text instructions describing the test exercise (Figure 3-7), and introduced tempo selection buttons that started the metronome at Tempo 1, 2, or 3 (50, 75, or 100 BPM) (Figure 3-8). This screen also included a self-assessment section with tempo-based checkboxes (Figure 3-9) and a button that advanced to the next test exercise (Figure 3-10).

#### 3.3.1 Usability issues identified in Prototype 1B

The two-screen design and lengthy text instruction was associated with increased reading and memory demands compared to the single-screen design in Prototype 1A.

Once on the second screen, the participants no longer saw the illustration and forgot details about the test exercise execution. This led to uncertainty about test exercise execution and about how to self-assess it (P5-P7). The stick-figure illustration's disappearance on the second screen caused frustration and loss of confidence while performing the test exercise. Participants wanted to verify their posture while performing the test exercise and experienced that they were unable to due to lack of visual cues.

The expression 'slow tempo' on the first screen and the three tempos on the second screen caused confusion making P5 wonder if and how they correspond to each other.

The phrase “touch the chair without sitting down” caused confusion for P6 who expressed it as being difficult to interpret.

P6 asked about arm use and expressed a need for more detailed instruction.

The phrase “on every other sound from the metronome” caused confusion about rhythm timing and resulted in P6 making more squats per sound than intended.

Multiple checkboxes caused confusion for P5 who wanted to check all completed tempos despite the instruction to choose only the highest completed tempo.

Moving between the two screens felt like starting a new test exercise for P5.

No chair stability guidance caused one near incident as the chair sled backwards during test exercise execution for P5. This revealed a need for further instructions regarding placement of the chair.

#### 3.3.2 Key design implications

After usability testing of Prototype 1B, the identified issues signaled a need for improvement in the next iteration (Usability phase 2).

The findings indicated that the two-screen layout combined with lengthy textual instructions increased cognitive load and disrupted continuity, suggesting a need for text length reduction and a unified single-screen instruction. The loss of the test exercise illustration on the second screen signaled a need for persistent visual cues to support posture verification, execution confidence, and self- assessment during the execution. Inconsistencies in terminology across screens—such as the relationship between “slow tempo” and the three numbered tempos—highlighted a need for clearer conceptual mapping and consistent language. Several instruction phrases were difficult to interpret, including descriptions of chair contact, arm placement, and rhythm timing, signaling a need for more explicit and unambiguous guidance. Confusion around multiple checkboxes indicated a need to simplify the self-assessment UI to clarify assessment criteria. Finally, the near incident involving chair instability revealed a need for visible safety guidance regarding chair placement during test exercise execution, as instructions provided earlier in the test were not reliably retained.

### 3.4 Prototype 2

Prototype 2 (Figure 4) addressed the main usability issues identified during Usability phase 1. It used a single-screen layout in which the instruction, metronome, illustration, and self-assessment were presented together to reduce cognitive load and prevent users from forgetting details between screens. A numeric progress marker (Figure 4-1) was used to present the participant's progression through the test. The illustration (Figure 4-2) remained visible throughout the execution to provide a consistent visual reference for movement. The text exercise instruction (Figure 4-3) was short, divided into clear paragraphs, and emphasized critical elements, such as “touch the chair lightly with your buttocks” and “3 consecutive squats for a tempo”, through bold formatting. To support intuitive interaction, the metronome slider (Figure 4-4) was presented vertically, with lower tempos at the bottom and higher tempos at the top. The self-assessment (Figure 4-5) was streamlined into a single numeric field in which participants entered the highest successfully completed tempo (as in Prototype 1A). Additionally, Prototype 2 included arm guidance and introduced a three-column layout to structure information more clearly and support test exercise execution.

#### 3.4.1 Usability issues identified in Prototype 2

The phrase “on every other beat of the metronome” caused confusion, as two (P8, P9) of the four participants performed the test exercise at half the intended tempo. P9 suggested the phrase “For each beat, you should either sit down or stand up.”

Due to the expression “Set the metronome to the desired tempo”, P8 was uncertain whether to test all tempos or choose one, leading to confusion: “It's unclear to me: there are three tempos to choose from—am I supposed to do all three, or do I choose the tempo I want?”.

P10 suggested placing the chair against a wall for stability “Good to have a chair against a wall so it doesn't slide away” and P11 expressed confusion about chair placement “Should the chair be placed against a wall … was that it?”.

Uncertainty if the test exercise was being correctly performed was expressed by P10 who wondered where the boundary between “sitting” and “touching” the chair with her buttocks is: “Where is the line between touching and sitting?”. This led to uncertainty during self-assessment, with P10 explicitly expressing her hesitancy: “I know at least that I wasn't sitting comfortably”.

#### 3.4.2 Key design implications

After usability testing of Prototype 2, the identified issues signaled a need for improvement in the next iteration (Usability phase 3).

The findings highlighted that rhythm-related instructions were insufficiently explicit, as the phrase “on every other beat of the metronome” led participants to misinterpret the intended movement timing and perform the test exercise at half the intended tempo. This signaled a need for more concrete phrasing that directly maps each beat to a specific movement. Ambiguity in the instruction to “set the metronome to the desired tempo” revealed uncertainty about whether all tempos should be tested or a single tempo selected, indicating a need to clarify test exercise structure and sequence. Safety-related uncertainty regarding chair placement further underscored a need for visible and specific guidance to support stable setup during execution. Finally, difficulty interpreting the boundary between “touching” and “sitting” on the chair introduced uncertainty both during test exercise execution and subsequent self-assessment, signaling a need for clearer articulation of execution criteria in the description of the test exercise to support appropriate test exercise execution and self-assessment.

### 3.5 Prototype 3A

Prototype 3A (Figure 5) addressed the main usability issues identified during Usability phase 2. The header and numeric progress marker (Figure 5-1) remained visible at the top of the screen. The primary text instruction was presented in a speech-bubble (Figure 5-2) attached to the simple stick-figure illustration, used earlier in Prototypes 1B and 2 (Figure 5-3), highlighting key movement instructions and reducing the need to read long text blocks. Additional text instructions (Figure 5-4) provided further guidance on test exercise execution and included a line encouraging the user to make a few attempts, to reassure users and promote learning through practice. The vertical metronome control (Figure 5-5), retained from Prototype 2, allowed participants to select among three predefined tempos (50, 75, and 100 BPM) and to start or stop the metronome. The self-assessment section (Figure 5-6) was simplified compared to Prototype 2, as the button “Save” was omitted. A navigation button (Figure 5-7) enabled participants to proceed to the next test exercise.

#### 3.5.1 Usability issues identified in Prototype 3A

Five participants tested Prototype 3A (P12–P16). While P13 completed the test exercise without expressing or exhibiting usability issues, the remaining participants encountered several challenges, as described below.

P12 found the metronome to be “the most challenging” element and did not understand how the tempo functioned, preferring instead a description of “gradually increasing tempo” rather than fixed tempo levels.

Two participants (P14, P16) completed a very large number of squats because they lost track of how many squats that should be performed, which may have inadvertently contributed to fatigue and poorer execution at higher tempos. One of these participants failed to locate the information and decided on her own to “just go for as long as I can manage” (P16).

Two participants did not position the chair against the wall until prompted, indicating that even though chair placement was addressed in the text instruction, it was not always acted upon (P15, P16).

Based on observations, P16 interpreted the details on the stick-figure as indicating that feet should be placed directly against the chair, making it physically impossible to touch the chair without sitting. P16 confirmed it by saying “I have that picture in my head… the feet are too close”. Incorrect positioning of the feet contributed directly to execution errors leading to shallow squats.

Two participants (P15, P16) adjusted their arm placement during the test exercise execution by holding arms on either the chest, down at their sides or on their knees. These improvised arm placements reflected uncertainty and lead to compensatory movements that influenced balance and squat depth. P15 explicitly expressed uncertainty about quality of her test exercise execution “I failed a bit at the beginning”.

The same participant (P15) had difficulty determining whether she was expected to perform all tempos sequentially or could choose freely where to start. This caused hesitancy and P15 verbalized it by saying: “I have no idea, I guess I'll start at 50”.

#### 3.5.2 Key design implications

After usability testing of Prototype 3A, the identified issues signaled a need for improvement in the next version (Usability phase 4).

The findings indicated that the metronome and tempo concept remained cognitively demanding for some participants, with fixed tempo levels being difficult to interpret. Loss of repetition-count awareness (required number of squats) led some participants to continue exercising beyond the intended number of squats, which signaled a need for clearer access to the required number of repetitions. Misinterpretation of stick-figure illustration led to incorrect squat depth, signaling a need for clearer visual guidance. Although chair placement against a wall was described in the text instruction, it was not consistently acted upon, indicating that safety-critical setup instructions required greater visibility. Observed execution deviations, such as arm adjustments made to maintain balance, highlighted a need for clearer guidance to support execution stability. Finally, uncertainty about whether tempos should be performed sequentially or could be freely chosen revealed a need to clarify the order of tempos to reduce hesitation and support confident test exercise execution.

### 3.6 Prototype 3B

Prototype 3B (Figure 6) addressed the main usability issues identified during Usability phase 2. The header and numeric progress marker (Figure 6-1) were visible at the top of the screen to support orientation and indicate progression through the test. A short instruction highlighting prohibited arm use (Figure 6-2) was presented in a speech-bubble attached to the stick-figure illustration. The simple stick-figure illustration (Figure 6-3), retained from Prototype 1B, depicted the squat movement. The main text instruction (Figure 6-4) described the test exercise and explained movement synchronization with the metronome sound using wording that differed from Prototype 3A. The vertical metronome control slider (Figure 6-5), retained from Prototype 2, allowed users to select among three predefined tempos (50, 75, and 100 BPM) and to start or stop the metronome. As in Prototype 3A, the self-assessment section (Figure 6-6) was simplified by omitting the previously included “Save” button, leaving a single input field for reporting self-assessed execution. A navigation button (Figure 6-7) enabled participants to proceed to the next test exercise. The overall layout remained identical to the layout tested in Prototype 2.

#### 3.6.1 Usability issues identified in Prototype 3B

Five participants tested Prototype 3B (P17–P21). While P21 completed the test exercise without expressing or exhibiting usability issues, the remaining participants encountered several challenges, as described below.

Two participants (P17, P19) explicitly commented that the prototype did not state how the arms should be positioned, only what users were not allowed to do with them. This led to hesitation and repeated re-reading as expressed by P17: “Does it matter if I hold my arms like this or like that? It doesn't say anything…”. P19 shifted arm placement during the execution to stabilize balance, suggesting uncertainty regarding proper execution criteria.

The starting position was unclear to P19, who was unsure whether the execution should begin from a standing or seated position.

P19 struggled with interpreting the boundary between touching and sitting on the chair “Was that enough touching? … It's kind of hard to interpret”.

There was a confusion regarding tempo selection and metronome meaning. Participants did not understand what the tempo values “50, 75, 100” represented, how to choose a starting tempo, or which rhythm should be followed. P17 tested only the middle tempo (75) because he did not understand that all three should be attempted. When the researcher asked P17 to repeat the test exercise after completing the whole test, he said that he “felt that was enough”. P20 started with 100 and worked backwards, indicating a misunderstanding of the progression logic and demonstrated entirely incorrect timing across all tempos tested. P17 experienced that he is the one to decide his own tempo “a certain pace … maybe every 3 s?” and after re-reading the instructions, he expressed uncertainty: “It shows 50, 75, 100 here … and I don't know what that means.” Other participants expressed similar misunderstandings about what the “desired tempo” referred to. P18 stated: “Set the metronome to the desired tempo. I don't know which tempo is desired. What does that mean?” Likewise, P19 asked: “Which tempo? I don't know what it says … should I set it to the lowest one?”

Losing track of the number of squats that should be performed occurred as another usability issue, as P17 lost all sense of how many squats he had completed, performing numerous squats without understanding when to stop.

Another observed usability issue was giving up after failed attempts. P19 abandoned further attempts after an unsuccessful trial. It indicates that the encouragement to repeat attempts is needed.

#### 3.6.2 Key design implications

After usability testing of Prototype 3B, the identified issues signaled a need for improvement in the next version (Usability phase 4).

An ambiguous arm-position guidance signaled a need for an explicit instruction that clearly states which arm placements are allowed, rather than only what is restricted. An unclear starting position signaled a need to clearly define the initial stance to avoid hesitation before test exercise initiation. Difficulty interpreting the boundary between touching and sitting signaled a need for more detailed instruction to support confident and correct execution and accurate self-assessment. Misunderstood tempo values and sequence order signaled a need to clearly explain what the tempo values represent and to clearly communicate the intended tempo progression. Loss of repetition-count awareness signaled a need to make completion criteria more visible such that users know when to end the test exercise. Finally, giving up after one failed attempt signaled a need to highlight that repeated attempts are expected and allowed.

### 3.7 Prototype 4

Prototype 4 (Figure 7) addressed the main usability issues identified during Usability phase 3, and those issues from Usability phases 1 and 2 that had not been previously addressed. As the tool was adapted for mobile use, a single-screen layout was no longer feasible. Instead, the content was distributed across screens with consistent navigation to maintain orientation and reduce cognitive load.

The header (Figure 7-1) was placed at the top of the screen to identify the current test exercise. A progress bar (Figure 7-2) visually displays the user's progress through the test exercise sequence, showing the percentage completed and the percentage remaining. Below the progress bar, an icon set (Figure 7-3) indicate the physical ability/ies being tested in the current test exercise (i.e., strength and coordination/speed) and the number of repetitions (i.e., 3×). The text instruction (Figure 7-4) provides a written description of how to perform the test exercise.

A bottom navigation panel (Figure 7-5) allows navigation to four sections: text description, video instruction, photo instruction, and self-assessment. Instructional videos filling up the full screen (Figure 7-6) were introduced to address recurring execution and self-assessment problems observed in earlier prototypes, where text-based instructions and static illustrations were insufficient to support correct timing, repetition awareness and execution. The video instructions included arrow-based navigation controls for switching between the introductory and tempo-specific videos. By providing synchronized visual and auditory guidance, the videos (Supplementary Videos S1–S4) were intended to reduce ambiguity in instruction interpretation and support more consistent execution and self-assessment.

A photo-based instruction filling up the full screen (Figure 7-7) replaced the earlier stick-figure illustrations to provide a clearer reference for body placement. In the Self-assessment section (Figure 7-8), users choose the highest tempo level at which they can perform the three squats before pressing the “Proceed to test exercise 7” button (Figure 7-9). An interaction walkthrough of Prototype 4 is provided in Supplementary Video S5.

#### 3.7.1 Usability issues identified in prototype 4

Transitioning to a mobile-based prototype introduced new interaction challenges, particularly regarding navigation within the video instruction section.

Difficulty or inability to locate tempo-specific instruction videos was the main challenge. All three participants (P22-P24) initially struggled to find the three tempo videos, and no one noticed the navigation arrows at first. Two participants eventually found the tempo videos but only after multiple attempts and extensive exploration (P22, P23). Although the underlying reason for this difficulty cannot be established based on the collected data, it may be understood in relation to the participants' prior interaction patterns with the self-assessment prototype, in which earlier test exercises had included only a single instruction video. Another possible contributing factor is that the videos were set to continuous auto-replay unless users actively selected another video or navigated away, potentially reducing attention to the navigation controls.

Additional challenge that emerged was performing test exercises while handling the mobile device. P23 explicitly stated that she was unsure how to look at the video and perform the test exercise simultaneously when holding the phone. This caused repeated switching between watching and performing, which disrupted execution flow.

Once the test exercise was completed, all participants (P22-P24) successfully navigated to the self-assessment section and understood the layout and required action, suggesting that navigation challenges were isolated to locating the tempo videos.

Compared to earlier prototypes, Prototype 4 was associated with less hesitation during test exercise execution, higher confidence in performing the test exercise, and fewer observable execution-related uncertainties. None of the participants testing this version expressed questions or hesitation regarding how the test exercise should be executed. The combination of text, photo, and video-based instruction appeared to support clearer interpretation of execution criteria, contributing to more confident and consistent execution.

#### 3.7.2 Key design implications

After usability testing of Prototype 4, the identified issues signaled a need for improvement in the future versions of the prototype.

The findings showed that transitioning to a mobile-based UI introduced new navigation challenges, particularly in locating tempo-specific instruction videos. The low discoverability of navigation arrows, which caused participants to overlook available content, signaled a need to make navigation controls more prominent and to better communicate the structure of the video sequence. Continuous auto-replay of videos further reinforced the assumption that no additional content was available, indicating that playback behavior should support progression rather than looping. Difficulties performing the test exercise while holding the mobile device highlighted a need for explicit guidance on device placement to support simultaneous viewing and execution. Importantly, once the test exercise was completed, participants navigated the self-assessment section without difficulty, suggesting that usability challenges were localized to video navigation component rather than the overall interaction flow.

### 3.8 Observed patterns related to self-assessment during test exercise execution

Across usability phases, participants were asked to self-assess their test exercise execution after completing the *Tempo-guided chair squat*. Independent of prototype version, several recurring patterns were observed in how participants performed these self-assessments.

First, the participants commonly expressed confidence in their self-assessments despite observable deviations in tempo adherence during execution. In multiple cases, participants verbalized uncertainty while performing the test exercise, for instance hesitating about squat depth, arm placement, or tempo cues, yet subsequently reported having completed the test exercise successfully. These judgments were often grounded in a general sense of completing the test exercise rather than explicit evaluation against defined execution criteria.

Second, the participants' self-assessments were strongly shaped by the information made salient in the UI. Due to self-assessment sections being focused on selecting “the highest completed tempo”, the participants tended to prioritize tempo completion in their judgments, while other execution-related aspects received less attention. Importantly, the misalignment between the participants' self-assessments and the execution criteria explicitly stated in the self-assessment sections across all tested UIs persisted throughout all prototype versions and was not addressed during the iterative design process. It became fully apparent only in the final cross-phase analysis.

Third, mismatches between the participants' self-assessments and researchers' assessments of the test exercise execution were closely associated with usability issues identified earlier in the results. For example, misunderstandings of tempo cues, loss of repetition-count awareness, and ambiguous execution criteria often co-occurred with mismatches in self-assessments. These observations suggest that the participants' assessments reflected how the test exercise was framed and supported by the UI rather than their actual execution quality.

Importantly, these observations are not presented as measures of self-assessment accuracy. Differences in instruction, interaction, and prototype fidelity across usability phases preclude comparative or quantitative evaluation. Instead, the findings illustrate how participants self-assessed their own test exercise execution under varying design conditions.

### 3.9 Overview of observed usability issues across iterations

To provide a comprehensive overview of all identified usability issues and their resolution across prototype versions, Supplementary Table S1 maps issue occurrence and status throughout the iterative development process. The table illustrates how usability issues were progressively addressed, partially mitigated, or, in some cases, re-emerged in subsequent versions.

Across usability phases, similar usability issues were grouped according to their type, resulting in eight overarching breakdown categories: Translating Timing Information into Movement Phases, Distinguishing Correct and Incorrect Execution States, Maintaining Awareness of Required Number of Repetitions, Persistent Visual Guidance During Execution, Safety-Critical Setup Information, Interface Consistency, Understanding Exercise-Relevant Concepts, and Defining Explicit Self-Assessment Criteria. The full mapping of these categories and their associated issues across prototypes is provided in Supplementary Table S1.

## 4 Discussion

This section is organized as follows. Subsection 4.1 conceptualizes physical ability self-assessment as a complex, layered interaction, framing the overall interactional demands placed on users in unsupervised contexts. Subsection 4.2 synthesizes observed usability issues into a set of transferable design principles, followed by a series of subsections (4.2.1–4.2.8) that elaborate on each principle in relation to specific interactional challenges identified across the different versions of prototypes. Finally, Subsections 4.3 and 4.4 address study limitations and directions for future work.

### 4.1 Physical ability self-assessment as a multi-layered interaction

This study examined how UI design supports or constrains users' ability to interpret, execute, and self-assess their test exercise execution in a digital physical ability self-assessment without supervision. Rather than evaluating physical ability or self-assessment accuracy, the focus was on identifying design-related usability breakdowns and synthesizing them into transferable design implications. By analyzing usability findings across successive prototype versions of a single, instructionally complex test exercise, the study clarifies the design principles required to support digital physical ability self-assessment.

As observed in this study, participants were required to coordinate several task layers simultaneously: interpreting test exercise instructions including proper placement of the feet, buttocks and arms, synchronizing movement with tempo cues, tracking the number of squats, assessing their own test exercise execution, and interacting with the UI. While individual elements were often understandable in isolation, integrating them during real-time execution proved demanding. These issues indicate that difficulties cannot be attributed to unclear wording or isolated UI flaws alone. Instead, physical ability self-assessment emerges as a cognitively and physically layered interaction that places sustained demands on user attention, memory, and judgment.

The complexity of the interaction observed in this study aligns with design theory describing performance load as the combined cognitive and physical effort required to accomplish a task, where increased load is associated with longer performance times and higher error rates (33). When guidance was not continuously available, participants relied on recall or subjective impressions, increasing the risk of execution errors and differences between their self-assessments and observed performance. From a design perspective, this underscores that digital physical ability self-assessments should be treated as complex interaction scenarios rather than simple instruction-following tasks, and that UI should be carefully designed to provide support throughout the task.

### 4.2 Synthesizing usability findings into design principles

To move beyond prototype-specific observations, we synthesize usability findings into a set of design principles (Table 2) describing what users must be able to perceive, monitor, and interpret during unsupervised digital physical ability self-assessment. Rather than framing issues as isolated problems, this synthesis positions them as indicators of essential design requirements within the studied context. These design principles are analytically derived from observed usability breakdowns and are not presented as validated design solutions. Moreover, these design principles capture insights from a relatively homogenous user group which implies that their applicability to more diverse populations require further empirical validation. In addition, given the small per-phase sample sizes, the identified usability issues may not represent the full usability problem space but rather reflect the more salient and recurring breakdowns observed across iterations.

**Table 2:** Design principles derived from this study for supporting unsupervised exercise execution and self-assessment in digital instructional UIs.

Observed usability issues	Corresponding design principle	Impact on exercise execution and self-assessment
Participants misinterpreted tempo cues (e.g., BPM values, “every other beat”), performed out-of-sync movements, or performed exercise in unintended tempo order.	Users must be able to translate timing information into specific movement phases while performing the exercise.	Reduces tempo execution errors and prevents exercise execution overestimation based on misunderstood timing cues.
Participants were uncertain about correct squat depth, the boundary between “touching” and “sitting”, and acceptable arm placement, leading to shallow squats and compensatory movements.	Users must be able to clearly distinguish correct and incorrect execution states through visual guidance.	Supports correct execution and enables users to judge exercise execution against explicit criteria rather than subjective impression.
Participants lost repetition-count awareness, were unsure when to stop, or performed too many squats, sometimes leading to fatigue or degraded execution.	Users must be able to continuously access to the required number of repetitions during execution.	Helps prevent executing too many repetitions, supports more consistent execution, and clarifies when the exercise is completed.
Exercise illustrations disappeared between screens, were misleading, or insufficiently supported posture verification during movement.	Users must be able to reference a sufficiently detailed visual instruction while performing the movement, not rely on memory or assumption.	Improves exercise execution consistency, reduces uncertainty, and increases alignment between intended and performed movement.
Participants overlooked or misinterpreted chair placement instructions, resulting in unstable chair positioning and near incident during exercise execution.	Users must be able to identify and follow safety-critical setup instructions during exercise execution.	Supports safe execution conditions and prevents execution disruptions.
Participants relied on interaction patterns learned in earlier exercises, leading to confusion when structure differed.	Users must be able to rely on consistent interaction structures across exercises or be explicitly informed when deviations occur.	Reduces confusion, search behavior, and misinterpretation of exercise structure.
Unfamiliar terminology increased cognitive load and caused misunderstandings.	Users must be able to understand exercise-relevant concepts using familiar language or personalized UI.	Improves comprehension and reduces early execution errors.
Self-assessment UIs focused only on tempo completion, ignoring execution criteria, leading participants to report success despite incorrect execution.	Users must be able to assess their exercise execution using the same execution criteria that define correct execution.	Reduces false confidence and supports more informed self- assessment by linking it to explicit execution criteria.

#### 4.2.1 Translating timing information into movement phases

Across prototypes, misunderstandings of tempo cues revealed the need to support translation of abstract timing information into concrete movement phases. Participants misinterpreted BPM values, struggled with concepts such as “every other beat”, and sometimes performed the test exercise in a different tempo sequence than intended, for example starting at a medium or high tempo rather than the prescribed low tempo.

From a design perspective, these findings indicate that tempo cues should be directly tied to distinct movement phases, rather than described in ways that require users to interpret timing relationships during execution. When users were required to mentally translate tempo descriptions into movement phases during execution, test exercise execution depended on recall and real-time interpretation, increasing cognitive performance load and contributing to sequencing errors. Established usability principles emphasize minimizing cognitive load and favoring recognition over recall in complex tasks, particularly when mental processing is combined with physical action (33). Accordingly, tempo guidance should externalize timing structure by aligning cues directly with recognizable movement phases during exercise execution.

#### 4.2.2 Distinguishing correct and incorrect execution states

Difficulties interpreting squat depth, arm placement, and acceptable execution demonstrated the importance of clearly differentiating correct and incorrect execution states. Participants were often uncertain about what constituted sufficient squat depth, where the boundary lay between “touching” the chair with their buttocks and “sitting”, and which arm placements were acceptable, leading to shallow squats and compensatory movements.

Several of these issues were linked to the availability and persistence of instructional material. When visual references disappeared between screens or lacked sufficient detail, participants relied on memory or assumptions, resulting in uncertainty about correct execution. From a signal-to-noise perspective, the absence or weakening of critical visual cues reduced the salience of relevant information and increased cognitive effort (33). These findings highlight the importance of making execution criteria perceptible and verifiable during movement, rather than requiring users to assess correctness only after completing the test exercise.

#### 4.2.3 Maintaining awareness of required number of repetitions

Loss of repetition-count awareness further highlighted the need for continuous access to the required number of repetitions during execution. Participants were sometimes unsure when to stop, executed too many squats, which led to fatigue that degraded execution toward the end of the test exercise. Consistent with recognition over recall principle in demanding contexts, continuous access to relevant information supports more stable execution and clearer awareness about completing the exercise.

#### 4.2.4 Persistent visual guidance during execution

Across the usability issues identified in the prototype versions tested in the study, visual guidance emerged as a primary enabler of successful execution. Textual instructions were often read and understood before movement began but were often forgotten or misremembered once execution started. Longer texts increased cognitive load and made it difficult for participants to retain and apply all relevant details during execution, consistent with prior research indicating that excessive informational complexity can overwhelm users and negatively affect task performance (33, 35, 36).

In contrast, the introduction of video-based instruction in the final usability phase allowed visual cues to remain accessible during preparation and execution. Participants actively referenced these cues to verify test exercise execution including proper placement of the feet and buttocks, timing, and movement. Static stick-figure illustrations provided limited support for nuanced execution and were often misinterpreted, whereas video-based instruction reduced hesitation and supported execution that closely aligned with the demonstrated movement. These observations align with prior research showing that video-based instructions are often preferred over static images (11) and can improve performance confidence (6). In usability terms, the videos functioned as external memory aids, reducing the burden placed on users' working memory and supporting recognition-based interaction (10).

These findings suggest that in physically demanding tasks, visual guidance functions not merely as instructional content but as an integral interactional resource that supports continuous regulation of movement during exercise execution.

#### 4.2.5 Safety-critical setup information

Safety-critical information, such as chair placement, was not made sufficiently prominent in the interface. Across prototype versions, participants expressed uncertainty about correct chair positioning, sometimes overlooked or did not act upon placement instructions, and in one case an unstable setup led to a near incident. This finding underscores the importance of ensuring that safety-related setup instructions remain visible, salient, and unambiguous during exercise execution, rather than being provided only in previous instructional material or outside the immediate test exercise context.

#### 4.2.6 Interface consistency

Interface expectations shaped by earlier interaction with previous test exercises in the prototype further influenced execution. Previous test exercises followed a consistent structure, involving a single instructional video. When the test exercise *Tempo-guided chair squat* deviated from this pattern by introducing multiple tempo-specific videos, participants appeared to rely on learned interface conventions, leading to confusion and difficulty locating relevant information. Both classic usability heuristics and general design principles emphasize consistency as a means of enabling knowledge transfer and reducing uncertainty when users encounter new or complex tasks (10, 33). Similar effects were observed when certain movement details, such as arm placement, were omitted after earlier test exercises had explicitly specified them.

This highlights the importance of consistency in UI design or, when deviations are necessary, explicit communication of structural differences.

#### 4.2.7 Understanding exercise-relevant concepts

Participants could not be expected to memorize and manage all exercise components simultaneously, including tempo concepts, required number of repetitions, interface interaction and how to execute as well as to self-assess the test exercise execution. This challenge was amplified by unfamiliar terminology such as “metronome” or numerical tempo values, which some participants misunderstood or confused with other test exercise execution elements. From a usability engineering perspective, the use of system-oriented or unfamiliar terminology increases learning demands and undermines the principle of using the users' own language to support comprehension (10).

These findings point toward the need for personalization. Substantial variation was observed in how participants experienced terminology, instruction formats, and informational density. Such variation aligns with usability research demonstrating that individual differences in prior knowledge, domain familiarity, and system experience can significantly shape interaction outcomes (10). Allowing the UI to be adjustable based on users' preferences and physical activity–related knowledge—such as preferred instruction format or terminology level—could reduce onboarding burden and support a more tailored user experience.

#### 4.2.8 Defining explicit self-assessment criteria

Self-assessment emerged as an interaction layer built on successful instruction interpretation, execution monitoring, and awareness of self-assessment criteria. Participants' assessments were strongly shaped by what the UI emphasized. As self-assessment UIs emphasized tempo completion while omitting other execution criteria, participants often reported successful test exercise execution despite observable deviations in observed performance. Although participants expressed confidence in their self-assessments, observational data revealed mismatches between the participants' self-assessment and researchers' assessment of the test exercise execution. In a real-world context, such discrepancies may influence how users interpret their performance and could affect subsequent training, monitoring, or rehabilitation decisions.

From a usability perspective, this reflects insufficient feedback about how the system interprets user performance. Usability engineering emphasizes that systems should continuously inform users about relevant states and interpretations, particularly in complex or safety-relevant tasks (10). When explicit support for monitoring execution quality is lacking, self-assessment relies on subjective impressions rather than explicitly defined criteria. This reinforces prior evidence that agreement between subjective and objective measures depends heavily on task design (30), while improvements in instructional clarity can reduce execution errors in self-administered physical tests (4, 25). Improvements in instructional clarity and persistent guidance, as observed in the final usability phase, therefore play a crucial role in supporting more consistent and informed self-assessment. Additionally, explicitly stating what constitutes successful completion is essential.

A notable aspect of the issue with the self-assessment criteria is that it remained unresolved across all prototype versions and became apparent only during the final cross-phase analysis. During usability testing, participants expressed confidence in their self-assessments and did not report difficulties, meaning the issue did not emerge as actionable within individual prototype versions. It was only when comparing self-assessments with observed execution across prototypes that a consistent pattern became visible: participants based their assessment on tempo completion while overlooking execution quality. This suggests that certain usability issues in complex interactions may remain latent in iterative UCD processes when they are not consciously perceived by users. To address this, iterative evaluations should be complemented with cross-phase analysis that examines patterns across prototypes and incorporates observational data beyond user feedback, as users may not be able to articulate all relevant usability issues.

### 4.3 Limitations

The study sample was dominated by participants with higher educational background and office-based work. As a result, the findings may not fully reflect the UI-related usability challenges experienced by the broader population of working-age adults, a heterogeneous group encompassing a wide variation in physical ability and physical literacy, digital literacy, everyday activity contexts and familiarity with digital exercise instructions. This limits the representativeness of the user group and constrains the generalizability of observed usability patterns beyond users with relatively high cognitive and technological resources. On the other hand, people with physical work backgrounds may have higher physical literacy and find exercise-based tasks easier, especially when it comes to performing the movements.

This study employed small user samples per prototype version within an iterative usability evaluation. Consequently, the findings should not be interpreted as an exhaustive inventory of all possible usability issues associated with the tested UIs. Prior research has demonstrated that usability problems vary substantially in their visibility, and that small-scale usability studies systematically underestimate the number of remaining, undiscovered problems, particularly when completeness is treated as an evaluation goal (37).

In the present study, however, the aim was not to achieve comprehensive discovery of all usability issues within a single test, but to identify design-relevant usability breakdowns that could inform successive refinement across evolving prototypes. The iterative structure of the study, in which design changes were introduced between usability phases, further limits the applicability of completeness-oriented sample-size assumptions, as each iteration altered the problem space under evaluation.

The tests were conducted in a controlled laboratory setting which offers practical advantages due to the high level of experimental control. However, field-based testing, such as in home environments for this type of technology, could yield more valid and realistic insights. The influence of the test settings in this study—lab settings—do not necessarily reflect those of the real use context. Several participants appeared visibly stressed during testing, possibly due to observation and the presence of researchers, which may have influenced their interaction with UIs. Conducting the tests independently at home, in a familiar environment and without an audience, might have resulted in different user behaviors and usability outcomes. For example, prior studies (4) have reported that home-based usability testing of an app-based self-administrable clinical tests of physical function revealed new usability issues although the app was tested twice in lab settings beforehand.

Despite this limitation, the controlled laboratory setting enabled detailed observation of execution errors and self-assessment reasoning that would have been difficult to capture in field settings, providing valuable insight into early-stage design-related challenges.

Furthermore, a limitation of this study concerns the shift from desktop-based prototypes (Prototypes 1–3) to a mobile-based prototype (Prototype 4). This change introduced differences in interaction conditions, including smaller screen size and the need to handle the mobile device during test exercise execution. These factors may have influenced how the participants interacted with the prototype. In addition, Prototype 4 introduced video-based instructions, providing continuous visual guidance during execution. At the same time, the mobile format also introduced new interaction challenges. The observed differences should therefore be understood as reflecting both the revised UI design and the mobile context, rather than the device format alone. A more controlled evaluation separating these factors would be needed to isolate their individual effects.

### 4.4 Future work

Building on the present results and insights from a parallel study, future work will involve developing a revised prototype that integrates the identified design requirements into a unified UI for a digital physical ability self-assessment tool. This prototype will be evaluated in a follow-up study to examine whether addressing the identified usability breakdowns improves execution quality and self-assessment quality under unsupervised conditions.

Given the observed mismatches between participants' self-assessment and researchers' assessment of the test exercise execution, future research in the broader field should prioritize self-assessment methods that integrate objective sensing technologies (e.g., wearable sensors or automated video analysis) with guided self-assessment, so that users receive feedback not only on exercise completion but also on execution quality. Building on evidence that wearable-derived measures of physical activity outperform self-report in terms of validity (34), such hybrid approaches represent one possible direction for addressing discrepancies between perceived and observed execution. However, the integration of such technologies falls beyond the scope of the present work.

In parallel, future work should explore how personalized UIs can better support diverse user preferences and needs, as tailored levels of guidance, feedback, and interaction may enhance usability, safety, and overall effectiveness of digital self-assessment tools. For instance, this could include experimenting with adaptive interfaces with varying types of instructional guidance (e.g., text-only, visual, audio-only, audiovisual) and evaluating their effectiveness in terms of the accuracy of alignment between users' self-assessment and their actual performance. Their effectiveness could also be evaluated in terms of task efficiency (e.g., reduced time and effort). In addition, users could be divided into groups (e.g., based on prior PA practices, digital and physical literacy) and examining how different user groups benefit from different UI configurations.

## 5 Conclusions

This study examined how UI design shapes users' ability to interpret, execute, and self-assess their execution of a digitally delivered physical ability test exercise under unsupervised conditions. Through iterative refinement of a single instructionally complex test exercise UIs, the findings show that physical ability self-assessment constitutes a multi-layered interaction rather than a simple instruction-following task. Users were required to coordinate interpretation of test exercise instructions, timing synchronization, repetition-count awareness, safety considerations, and self-assessment simultaneously during execution.

Across prototype versions, usability breakdowns consistently emerged when users had to rely on memory, inference, or subjective judgment. Misinterpretations of tempo cues, unclear execution criteria, loss of repetition-count awareness, and misalignment between execution and explicit self-assessment criteria contributed to discrepancies between participants' self-assessment and researchers' assessment of the test exercise execution. These issues could not be resolved through clearer wording alone but required design solutions that externalized exercise structure and supported continuous access to execution criteria.

By synthesizing findings across design iterations, the study contributes a set of transferable design principles describing what users must be able to access, monitor, and interpret during unsupervised physical ability self-assessment. While grounded in a specific self-assessment context, these design principles may be applicable to exercise-based instructional UIs more broadly, where correct execution and self-assessment must be achieved without professional supervision, although their generalizability to other exercises and contexts requires further validation.

This study further contributes to UCD and user experience design by extending established usability principles to multi-layered interaction contexts involving simultaneous cognitive and physical performance. The findings show that principles such as recognition over recall, feedback, and consistency must be operationalized as continuous, execution-integrated guidance rather than as static UI properties.

Importantly, as the study was conducted with a relatively homogeneous and highly educated participant sample, the identified usability challenges may underestimate those encountered in more diverse real-world populations. In addition, due to the small per-phase sample sizes typical of iterative usability testing, the reported usability issues may not represent the full usability problem space and may primarily reflect more salient or recurring breakdowns. Future research is therefore needed to validate and extend these design principles across broader user groups. This could involve implementing the design principles in concrete prototypes and evaluating them through usability testing with participants varying in digital and physical literacy, education, and professional background.

## Data Availability

The datasets presented in this article are not readily available because they consist of video recordings that contain identifiable participant information and cannot be anonymized without compromising participant privacy. Requests to access the datasets should be directed to agnieszka.jaff@mdu.se.
