# Supplementary material for: Designing interfaces for digital physical ability self-assessment: a user-centered iterative approach
Source: Front Digit Health. 2026 Jun 25;8:1815892. doi: 10.3389/fdgth.2026.1815892 (PMC13346045; doi:10.3389/fdgth.2026.1815892)
Supplement: Supplementary file 1 [file Table1.docx]

**Supplementary Table 1. Mapping of identified usability issues and their resolution across prototype versions. Symbols indicate the status of each usability issue in each prototype version: ● = observed; ◐ = addressed but still present; ✓ = addressed and resolved (not observed); ↺ = reappeared after prior resolution; – = not observed.**

| **Breakdown category** | **Specific usability issue** | **Prototype 1A** | **Prototype 1B** | **Prototype 2** | **Prototype 3A** | **Prototype 3B** | **Prototype 4** |
| --- | --- | --- | --- | --- | --- | --- | --- |
| **Translating Timing Information into Movement Phases** | Confusion – how “slow tempo” from Screen 1 corresponds with the three tempos on Screen 2 | – | ● | ✓ | – | – | – |
|  | Every other sound' timing misunderstood. | – | ● | ✓ | – | – | – |
|  | Phrase 'on every other beat' caused confusion. | – | – | ● | ✓ | ✓ | – |
|  | 'Set the metronome to desired tempo' unclear: choose one tempo or test all? | – | – | ● | – | – | ✓ |
|  | Misunderstood tempo values and sequence order. | – | – | – | – | ● | ✓ |
|  | Uncertainty about tempo sequence order. | – | – | – | ● | – | ✓ |
|  | Confusion from multiple tempo-based checkboxes. | – | ● | ✓ | – | – | – |
| **Distinguishing Correct and Incorrect Execution States** | Unclear distance/depth due to lone chair illustration resulting in shallow squats. | ● | – | ✓ | – | – | – |
|  | Unclear arm placement. | ● | ● | ✓ | ↺ | – | ✓ |
|  | Phase 'touch the chair without sitting' unclear. | – | ● | ◐ | ✓ | ↺ | ✓ |
|  | Stick-figure implied incorrect foot placement. | – | – | – | ● | – | ✓ |
|  | Unclear starting position. | – | – | – | – | ● | ✓ |
| **Maintaining Awareness of Required Number of Repetition** | Phrase 'three full laps' unclear. | ● | – | ✓ | – | – | – |
|  | Loss of repetition-count awareness | – | – | – | ● | ● | ✓ |
| **Persistent Visual Guidance During Execution** | Loss of illustration and exercise instruction on second screen caused uncertainty about exercise execution and self-assessment. | – | ● | ✓ | – | – | – |
|  | High cognitive load from two-screen layout and lengthy text instructions. | – | ● | ✓ | – | – | – |
|  | Assuming that the looping intro video was the only available video. | – | – | – | – | – | ● |
|  | Giving up after failed attempt. | – | – | – | – | ● | ✓ |
|  | Difficulty exercising while holding phone. | – | – | – | – | – | ● |
| **Safety-Critical Setup Information** | Uncertainty about safe chair positioning and unstable test exercise execution due to incorrect chair placement. | – | ● | ◐ | ◐ | ✓ | ✓ |
| **Interface Consistency** | Navigation between screens felt like starting a new test exercise. | – | ● | ✓ | – | – | – |
|  | Difficulty or inability locating tempo videos. | – | – | – | – | – | ● |
| **Understanding Exercise-Relevant Concepts** | Phrase ‘squat’ unclear. | ● | – | ✓ | – | – | – |
|  | Metronome difficult to understand. | – | – | – | ● | – | ✓ |
| **Defining Explicit Self-Assessment Criteria** | Self-assessment focused on tempo completion while execution criteria remained implicit, leading to reported success despite incorrect execution. | ● | ● | ● | ● | ● | ● |
