# Supplementary material for: Designing interfaces for digital physical ability self-assessment: a user-centered iterative approach
Source: Front Digit Health. 2026 Jun 25;8:1815892. doi: 10.3389/fdgth.2026.1815892 (PMC13346045; doi:10.3389/fdgth.2026.1815892)
Supplement: Supplementary file 2 [file Datasheet1.docx]

Supplementary Material 1

# Test Scripts used across the four usability phases (English Translation)

This appendix presents only the segments of the original usability test scripts that are directly relevant to the usability issues analyzed in the paper. The full scripts included several additional tasks and instructions, but since the study focuses specifically on the test exercise examined in the Results section, only the corresponding parts are reproduced here.

## Prototype 1A and 1B – Test Script

BRING WITH YOU

(…)

• Computer prepared with a metronome (preferably a separate browser window with tabs for each tempo; use Google’s built-in metronome function: <https://share.google/eOIuEcBc6tugSJuX4>

Tempos needed:

o Test exercise 10: 50, 75, 100

WELCOME (DO NOT SHOW ANYTHING ON THE COMPUTER SCREEN AT THIS POINT)

Hello, and thank you for participating in the PRE-fall project, where we are developing a digital self-test for fall risk among adults of working age.

The purpose of the self-test is to assess different parameters related to fall risk. You have previously received an information letter – we hope that you have read it and wish to participate in today’s test of a very basic prototype of a digital self-test.

It is important to remember that we are not testing you, but rather the prototype.

We will walk through the various views that you will see, and we ask you to think aloud today. We may also ask some questions during the test.

Because we want to focus on the testing with you and create as natural a test situation as possible, your participation will be filmed from behind. This allows us to minimize note-taking today and enables later analysis and comparison of how different people interact with one of the two prototypes.

Do you have any questions before signing the consent form?

Great, then I’ll start the basic prototype. I will also start the recording and make sure the camera is directed at the computer screen.

The recording is now running.

I’d like to remind you that the interface you are about to see is not interactive.

You will therefore need to tell me out loud where you would want to click or type.

(…)

Prototype 1A — Test Exercise 10

Think aloud and tell me what I should do.

(…)

Prototype 1B — Test Exercise 10

How many test exercises have you performed now?

Think aloud and tell me what I should do.

(…)

## Prototype 2 – Test Script

BRING WITH YOU

(…)

• Computer prepared with a metronome (preferably a separate browser window with tabs for each tempo; use Google’s built-in metronome function: https://share.google/eOIuEcBc6tugSJuX4

Tempos needed:

o Test exercise 10: 50, 75, 100

WELCOME (DO NOT SHOW ANYTHING ON THE COMPUTER SCREEN AT THIS POINT)

Hello, and thank you for participating in the PRE-fall project, where we are developing a digital self-test for fall risk among adults of working age.

The purpose of the self-test is to assess different parameters related to fall risk. You have previously received an information letter – we hope you have read it and wish to participate in today’s test of a very basic prototype.

It is important to remember that we are not testing you, but the prototype.

We will walk through the various views you will see. We therefore ask you to think aloud today. We may also ask some questions during the test.

Because we want to focus on testing with you and create as natural a test situation as possible, your participation will be filmed. We will try to avoid filming your face, but we cannot guarantee it. The purpose is to enable later analysis and comparison of different people’s interaction with the prototype.

Do you have any questions before signing the consent form?

Great, then I’ll start the basic prototype. I will also start the recording and make sure the camera is directed at the computer screen.

The recording is now running.

I’d like to remind you that the interface you will see is not interactive.

I also want to emphasize that you will be seeing a very stripped-down version of the self-test – you don’t need to think about colors and such.

You will need to tell me out loud where you would want to click or type.

(…)

Test Exercise 10 — METRONOME

## Prototype 3A and 3B – Test Script

BRING WITH YOU

(…)

• Phone with prepared metronome (the iPhone Appstore app is called Smart Metronome and Timer and shows 60 with three bars in the picture) (…)

WELCOME (DO NOT SHOW ANYTHING ON THE COMPUTER SCREEN AT THIS POINT)

Hello and welcome.

(…)

Your participation today has two purposes:

1. You will perform the self-test using a basic prototype and receive a result.

2. You are important for the further development of our digital self-test.

It is important to remember that we are not testing your understanding of the content in the basic prototype. You are extremely important for helping us improve the information in it.

We will walk through the prototype’s different views, and we ask you to think aloud as you see the views and perform what you are prompted to do.

Please tell me out loud where you would want to click or type.

Then I will start the basic prototype – the self-test. I will also start the recording and ensure that the camera is directed at the computer screen.

The recording is now running.

## Prototype 4 – Test Script

(…)

TO DO BEFORE THE PARTICIPANT ARRIVES

(…)

• Prepare the phone with the prototype (turn off screen rotation and notifications)

(…)

WELCOME

Hello and welcome.

(…)

Today you will use an interactive prototype on a mobile phone, and I ask you to think aloud as you look at the screen and perform what you are prompted to do. Please tell me out loud where you would want to click or type. You will perform a self-test of your fall risk and later receive a result.

It is important to remember that I am not testing your understanding of the content in the prototype. You are extremely important for helping improve the information in it. Please make sure to say what you do not understand.

Try to act as if you are alone in the room. I will only intervene if I notice a risk of injury due to incorrect performance of the exercises, or if the prototype does not function as it should. I will also ask you to express aloud how you assess your performance after each exercise so that I can note this.

If you get completely stuck, explain why and ask to move on to the next exercise.

(…)

Then I will start the prototype – the self-test on the phone.

I am starting a screen recording on the phone.

The recording is now running.
